# Efficient gaze stabilization during video Active Gaze Shift Test (vAGST) in bilateral vestibulopathy

**DOI:** 10.3389/fneur.2024.1509762

**Published:** 2024-12-12

**Authors:** Christian Van Nechel, Ulla Duquesne, Michel Toupet, Charlotte Hautefort

**Affiliations:** ^1^Dizzy-Care - Clinique des Vertiges, Brussels, Belgium; ^2^Institut de Recherche Oto-Neurologique (IRON), Paris, France; ^3^Centre Hospitalier Interrégional Edith Cavell, Brussels, Belgium; ^4^Centre d’Explorations Fonctionnelles Otoneurologiques, Paris, France; ^5^Institut Pasteur, AP-HP, CHU Lariboisière, Service ORL, Paris, France, CMHP, CRMR VERTICO, Fondation Pour l'Audition, IHU reConnect, Université Paris Cité, Paris, France

**Keywords:** bilateral vestibular deficit, vestibulo-ocular reflex, active gaze shift, gaze stabilization, predictive perception, efference copy, vestibular rehabilitation, video head impulse test

## Abstract

**Introduction:**

While most head movements in daily life are active, most tools used to assess vestibular deficits rely on passive head movements. A single gain value is not sufficient to quantify gaze stabilization efficiency during active movements in vestibular deficit patients. Moreover, during active gaze shifts, anticipatory mechanisms come into play. Our aim was to explore the extent to which previously described compensatory mechanisms are employed in patients with bilateral vestibular loss.

**Methods:**

We used a Video Head Impulse Test (vHIT) to simultaneously record eye and head movements during a video Active Gaze Shift Test (vAGST). Thirty-eight patients with bilateral vestibular deficits and 61 control subjects were tested.

**Results:**

Despite impaired performance on caloric tests and vHIT, most patients exhibited normal gaze stabilization (gain = 0.92 ± 0.1) during active gaze shifts up to a head speed (‘stall speed’) of approximately 140 ± 60°/sec, compared to 280 ± 65°/sec in controls. Our results indicate that BVD patients spontaneously adopt a head speed during active horizontal movements that significantly improves gaze stabilization compared to passive movements. The stall speed correlates with the spontaneous head speed typically adopted by BVD patients and controls in daily activities. As a consequence of the reduction in head speed and corrective saccades, patients also showed an increased delay in gaze stabilization (413 ± 105 ms in BVD patients versus 358 ± 82 ms in controls) at the end of the gaze shift, which might become disabling for certain tasks.

**Discussion:**

Recent model suggests that compensatory eye movements, which stabilize gaze during the counter rotation phase of active gaze shifts, are predictive in nature. vAGST is not designed to provide an etiological diagnosis but rather a functional assessment of the patient’s ability to generate predictive eye movements that compensate for vestibular sensor deficits. Understanding the quality of the patient’s sensory predictions can also shed light on vestibular symptoms, even in cases where no vestibular sensor deficit is detected. This suggest that quality of life and oscillopsia questionnaires should distinguish between predictable and unpredictable movements.

## Introduction

In daily life, most head movements are active, involving coordinated head and eye movements to rapidly shift the direction of gaze (1–3). These gaze shifts, which redirect the line of sight and aim the fovea at different targets of interest in the environment, require efficient gaze stabilization, a process largely driven by the vestibular system. The current clinical assessment of vestibular function typically includes caloric tests, rotational tests, the video head impulse test (vHIT), passive dynamic visual acuity, and cervical and ocular vestibular evoked potentials. Notably, all these examinations use passive stimulation of the vestibular sensors. However, the mechanisms underlying gaze stabilization during passive movements may differ from those engaged during active movements, both in healthy individuals and those with vestibular deficits (4). Chen (5) showed significant degradation of DVA during gaze shifts in patients with unilateral vestibular deficit compared to control subjects but without comparison with the results obtained during passive movements.

**Figure 1:**
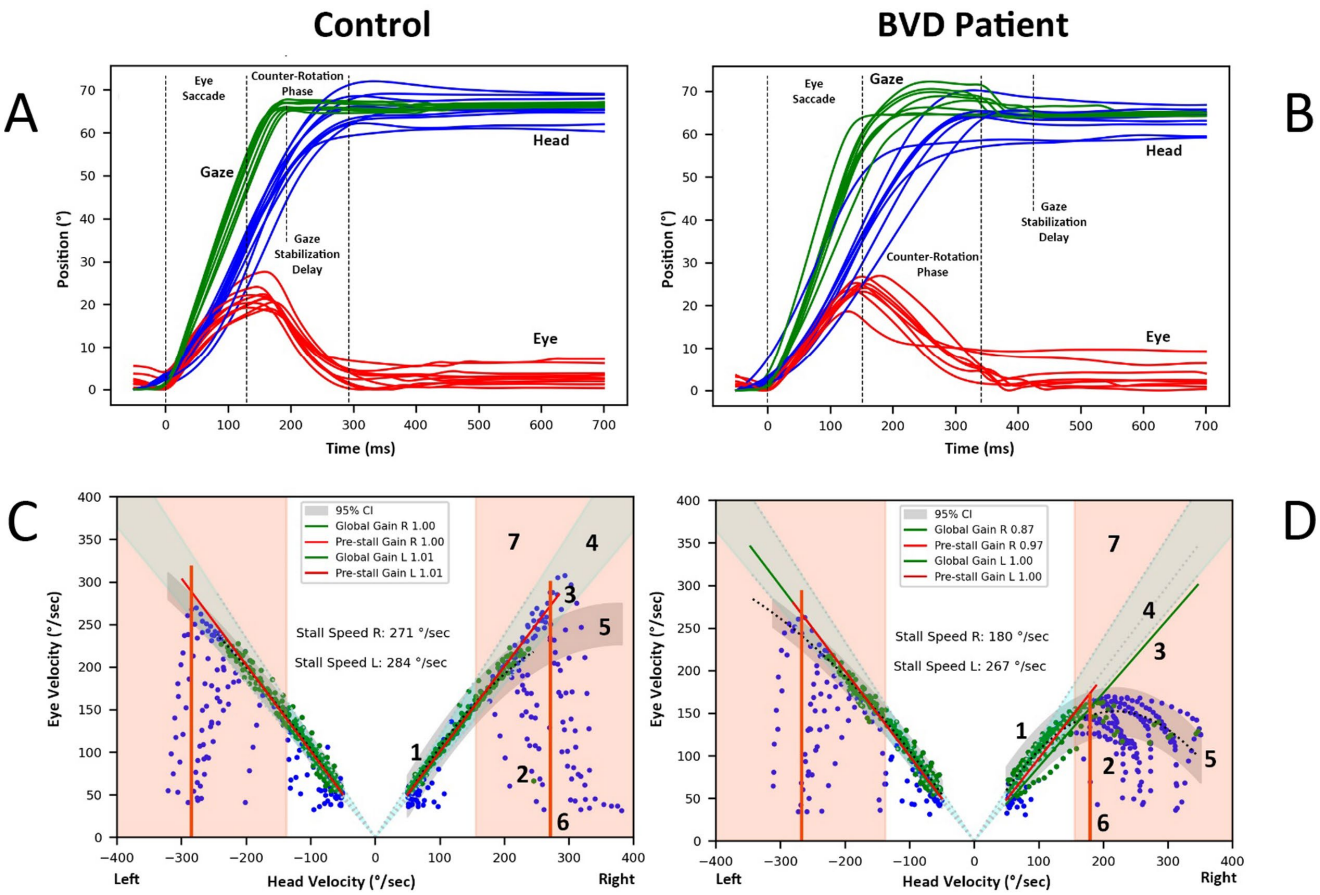
**(A)** Recordings of head-free rightward gaze saccades elicited by target steps of 60° in a normal subject. The eye performs an initial saccade of approximately 20–30°, followed by a counter-rotation in the opposite direction of the head movement, thereby stabilizing gaze. The end of the final corrective saccade marks the gaze stabilization delay. **(B)** Similar recording in a BVD patient with asymmetric vAGST, normal on the left side, despite profound bilateral caloric deficit. **(C,D)** Analysis of eye and head velocities (degrees/second) during a gaze shift in the same control **(C)** and BVD patient **(D)** 1. Green points represent the data used to estimate the overall gain and pre-stall gain. Pre-stall gain are computed on points below the stall speed (item 6 of this figure). 2. Blue points, which are excluded, primarily reflect the eye’s acceleration phase as it attempts to match the head’s velocity. 3. The Theil-Sen linear regression applied to the selected points defines the gain during the gaze stabilization phase of the gaze shift. 4. The area of normal gain limits is shown as 95% confidence interval. 5. Polynomial regression of the selected points, along with the prediction interval (grey area), illustrates the data's relationship. 6.The stall speed for rightward gaze shifts is identified based on the highest head velocity point within the intersection of the gain confidence interval (point 4) and the prediction interval (5). Data points where head speeds are below 50°/second or eye speeds are below 30°/second are excluded. 7. The area of normal gaze stabilization delay limits is shown as 95% confidence interval.

During gaze shifts, head movement usually begins simultaneously with, or slightly after, eye movement, depending on factors such as the ability to predict the position of the visual target (6–9). The eyes, however, reach the target more quickly and must then be stabilized while the head continues to move. In both healthy individuals and patient with bilateral vestibular deficit (BVD), the eyes generally begin to reverse direction at the end of the saccade during the counter-rotation phase, compensating for ongoing head movement (Figures 1A,B). This stabilization was considered to be mainly mediated by the vestibulo-ocular reflex (VOR) in intact monkeys (10) and normal human subjects (11). During gaze shifts, the VOR is inhibited until the end of the saccade, after which it reactivates to ensure post-saccadic gaze stabilization (12, 13). As a result, eye movements in healthy subjects are nearly perfectly compensatory for both passive and active head movements. Conversely, Bizzi (1) and Dichgans (14) demonstrated that recovery of ocular stability following bilateral labyrinthectomy in monkeys is a complex process. It involves centrally programmed compensatory eye movements, recalibration of the saccadic and head motor systems, and, to a lesser extent, an increase in the gain of the cervico-ocular reflex.

Few studies, with small sample sizes, have explored the counter-rotation phase in patients with bilateral vestibular deficit. Kasai et al. identified three distinct compensatory behaviors for eye-head coordination in three BVD patients (15). In Maurer’s study, the pooled results from six patients revealed that average gaze stability was comparable to that of controls 40 ms after the end of the eye saccade, though with greater variability and numerous inappropriate post-saccadic slow eye movements (8). Notably, post-saccadic eye drifts in the reverse direction were frequently observed when patients performed saccadic gaze shifts without concomitant head movement, suggesting the use of a pre-programmed, learned compensatory movement. Saglam (16) demonstrated that patients can better stabilize gaze during active head movements than passive ones, likely through feedforward mechanisms, though proprioception alone is insufficient. More recently, Haggerty et al. (17) proposed a model of gaze stabilization during planned head movements, involving pre-programmed eye movements (PPEM). They suggested that PPEM, in conjunction with the VOR, are used by both healthy and lesioned individuals to achieve gaze stability during active movements.

Recent models conceptualize the brain as an active predictive processor rather than a passive receiver of sensory data. It continuously predicts sensory inputs by generating and updating hypotheses through recurrent hierarchical processing (18). This perspective suggests that symptoms emerge from a mismatch between sensory predictions and actual sensory information (19). These predictive models also imply that the organism seeks to confirm its predictions through actions, which are adjusted based on discrepancies between expected and actual sensory inputs (20). This suggests that rehabilitation can not only enhance vestibular compensation but also reduce symptoms by optimizing sensory predictions.

Our aim is to investigate head-eye coordination in patients with bilateral vestibular deficit (BVD) and the extent to which they used the previously described compensatory mechanisms, as well as the conditions under which these mechanisms are effective. To that end we developed a simple test: the video Active Gaze Shift Test (vAGST).

## Materials and methods

### Patients with BVD

Thirty-eight subjects with bilateral vestibular deficit (BVD) (26 females, 12 males; mean age: 57.9 ± 15.7 years) were evaluated across three vestibular expertise centers. The study protocol received approval from the French Ethical Committee (*Comité de Protection des Personnes de la Région Ouest I, ID-RCB: 2022-AO1513-40*). Participants were recruited through the Association Française de Vestibulopathie Bilatérale (AFVB) and, as such, had all been previously diagnosed by a comprehensive battery of neuro-otological tests conducted at the *Centre d’Explorations Fonctionnelles Oto-Neurologiques* (*Paris*), the *CRMR VERTIco, University Paris Cité, Lariboisière Hospital APHP* (*Paris*), or the *Clinique des Vertiges* (*Brussels*).

The BVD had been present for an average of 12.8 ± 7.9 years prior to inclusion in the study. At the time of testing, all patients had adapted to their vestibular loss, which had a moderate functional impact on their daily lives. Despite this adaptation, they reported experiencing oscillopsia and imbalance in low-light conditions. None of the BVD patients had hearing loss or associated neurological symptoms, except one CANVAS patient.

The diagnosis of BVD was established based on standard otoneurological examinations, including bithermal caloric testing (irrigation of the left and right auditory canals with water at 44°C and 30°C), the video head impulse test (vHIT), and vestibulo-ocular response measurements during a pendular test on a rotating chair. In some patients, saccular and utricular functions were also assessed by recording cervical vestibular-evoked myogenic potentials (cVEMPs) over the sternocleidomastoid muscles and ocular vestibular-evoked myogenic potentials (oVEMPs) over the inferior oblique muscles, respectively. All patients met the Bárány Society criteria for bilateral vestibulopathy (21).

All patients exhibited reduced caloric test responses. The sum of the maximal peak velocities of the slow-phase caloric-induced nystagmus for warm and cold water stimulation was 1.1 ± 1.16°/s (mean ± SD) for the left ear and 0.89 ± 1.2°/s for the right ear. The mean VOR gains, as measured by vHIT (Otometrics – Natus), were significantly reduced (right horizontal canal: 0.34 ± 0.28; left horizontal canal: 0.28 ± 0.2). Notably, five patients had vHIT gains greater than 0.6 on at least one side but exhibited significantly impaired caloric test results (sum of the maximal peak velocities of the slow phase <6°/sec) on both sides.

The data from the BVD patients were compared with those from 61 control subjects (mean age: 48.7 ± 18.2 years; 41 females, 20 males). All control subjects were free from known vestibular and neurological disorders.

### Data acquisition

Each subject was instructed to alternately fixate on two fixed targets located 30° on either side of a midpoint, positioned 140 cm in front of them in a normally lit environment. The subjects were asked to perform a combined head and eye movement to shift their gaze between the targets, mimicking the speed of head movement typically used when checking for traffic before crossing a street. This speed was self-determined by the subjects, reflecting a natural rapid movement of daily life. While the speed was freely chosen, the examiner instructed the subjects to change fixation points approximately every second. Subjects were also asked to avoid blinking during the movement. Head and eye velocity data were recorded using the VOR recording option of the vHIT Otometrics – Natus system. After system calibration, head and eye movement data were recorded for 20 s at a sampling rate of 247 frames per second. The collected data were then exported for analysis using software specifically designed by one of the authors (CVN).

### Data analysis

Figures 1A,B illustrate the position of eye, head, and gaze (sum of eye and head postions) during a 20-s vAGST shift recording to the right in a normal and BVD subject, showing the eye saccade followed by the counter-rotation phase and the gaze stabilization delay after the corrective saccades. A Savitzky–Golay algorithm (third-degree polynomial) was employed to identify inflection points on the head and eye velocity plots, marking the beginning and end of eye saccades, head movements, and gaze shifts. These parameters were then used to determine the maximum velocities of saccades and head movements. Corrective saccades were identified within 900 ms after the start of the gaze movement, with the last identified saccade defining the gaze stabilization delay. In the absence of a corrective saccade, this delay was defined by the end of the gaze movement.

During the combined head and eye movement, the gaze typically reach the target at the end of an eye saccade. As the head continues to move, the eyes counter-rotate in the opposite direction of the head to stabilize the gaze on the target. We defined a global gain value as the ratio between eye speed and head speed during this counter-rotation phase. However, not all points corresponding to opposing eye and head speeds were included in this calculation. Specifically, points during the phase where the eye accelerated in the opposite direction to match the head speed were excluded (reference (2) in Figures 1C,D). Points were retained once the eye reached its maximum speed in this direction and while the head speed remained greater than 50°/second, to minimize the impact of potential ocular pursuit in the gain calculation (reference (1) in Figures 1C,D). Points where eye speed exceeded head speed were eliminated, as they corresponded to catch-up saccades. A Theil-Sen linear regression applied to the retained points determined the global gain value (reference (3) in Figures 1C,D).

We defined the stall speed as the maximum head speed at which gaze stabilization was possible (reference (6) in Figures 1C,D). This value was established based on the highest head speed point within the confidence interval of gain normative data (mean ± 2 SD) and within the prediction interval of a second-degree polynomial regression of the retained points for the overall gain calculation (reference (5) in Figures 1C,D).

As stabilization is not achieved with every gaze shift, a pre-stall gain was calculated using Theil-Sen linear regression on points where head speed was lower than the stall speed. A minimum of 10 points was required to calculate this gain, which indicates the consistency of gaze stabilization up to this speed. Its value decreases if eye speed is frequently lower than head speed.

A head motion ratio (HMR) was defined as the proportion of the head movement’s amplitude in the gaze shift, calculated as the sum of the eye and head movements.

Statistical analysis was performed using Statistica software. Comparisons between patients with bilateral vestibular deficit (BVD) and control subjects were made using the non-parametric Mann–Whitney test.

## Results

**Figure 2:**
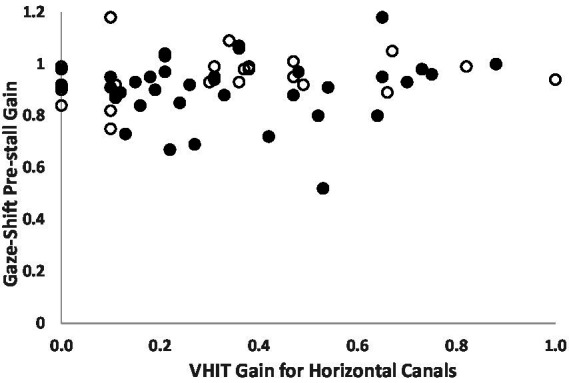
Comparison of each right and left gains measured by vHIT of the horizontal canals and the pre-stall gains during Gaze-Shift in BVD patients (*n* = 38). Solid black dots represent patients whose spontaneous head velocity during Gaze-Shift falls within the speed range used for vHIT (>150°/second). White dots represent patients with spontaneous head velocity < 150°/sec. The gains measured during the Gaze-Shift are significantly higher than those from vHIT, even at comparable head velocities. Note: BVD patients with gains > 0.6 have a profound deficit on caloric tests.

Table 1 shows the results of vAGST parameters in BVD patients and controls. The pre-stall gains in BVD patients were 0.9 ± 0.12 (right) and 0.95 ± 0.09 (left), significantly lower (*p* < 0.01) than the pre-stall gains of control subjects (1.05 ± 0.07 right, 1.04 ± 0.06 left). Only one patient with a vHIT gain <0.6 for the horizontal canals did not have a higher pre-stall gain during vAGST. There is a significant improvement between the pre-stall gains during vAGST in BVD patients and the gains measured during passive movements at vHIT (Figure 2). The black dots represent gains in patients who adopted a spontaneous head speed greater than 150°/sec, similar to the speeds induced during passive vHIT movements. These gains were significantly higher (mean difference for head velocities >150°/sec: 0.59 ± 0.32) than the horizontal vestibulo-ocular reflex (VOR) gains recorded during passive vHIT movements (right = 0.34 ± 0.28; left = 0.28 ± 0.2). The absence of correlation (*r* = 0.1, *p* > 0.05) indicates that vHIT and gaze-shift measurements involve distinct mechanisms. The overall gains in BVD patients during active movements were 0.78 ± 0.2 (right) and 0.83 ± 0.2 (left), significantly lower (*p* < 0.01) than those of control subjects (1.04 ± 0.07).

**Table 1:** Comparative analysis of the parameters studied in BVD patients and control subjects.

	Controls		Patients		Mann-Withney test
	Mean (s.d.)	Median	Mean (s.d.)	Median	*p*
Age	48.7 (18.2)	47.7	57.9 (15.7)	62.2	0.011
Stable gaze delay right (ms.)	359 (102)	354	422 (109)	398	< 0.01
Stable gaze delay left (ms.)	358 (63)	350	404 (100)	387	0.021
Right gaze amplitude (deg.)	61 (15)	62	53 (6)	53	< 0.01
Left gaze amplitude (deg.)	59 (18)	59	47 (7)	48	< 0.01
Right saccade amplitude (deg.)	23 (7)	23	23 (7)	23	n.s.
Left saccade amplitude (deg.)	22 (6)	21	21 (5)	20	n.s.
Right saccade velocty (deg./sec.)	372 (87)	353	363 (78)	360	n.s.
Left saccade velocty (deg./sec.)	364 (79)	344	380 (94)	356	n.s.
Global gain right	1.04 (0.07)	1.04	0.78 (0.20)	0.88	< 0.01
Global gain left	1.04 (0.07)	1.03	0.83 (0.21)	0.89	< 0.01
Pre-stall gain right	1.05 (0.07)	1.04	0.90 (0.12)	0.92	< 0.01
Pre-stall gain left	1.04 (0.06)	1.03	0.95 (0.09)	0.95	< 0.01
Head velocity right (deg./sec.)	289 (81)	281	186 (79)	170	< 0.01
Head velocity left (deg./sec.)	268 (64)	265	185 (86)	155	< 0.01
Stall speed right (deg./sec.)	295 (69)	286	133 (50)	124	< 0.01
Stall speed Left (deg./sec.)	266 (65)	263	142 (69)	124	< 0.01
Head mover ratio right	0.94 (0.22)	0.96	0.81 (0.25)	0.81	< 0.01
Head mover ratio left	1.03 (0.84)	0.91	0.77 (0.23)	0.75	< 0.01

The right and left stall speeds in BVD subjects were 133 ± 50°/sec and 142 ± 72°/sec, respectively, significantly lower (*p* < 0.01) than those of control subjects (295 ± 69°/sec and 266 ± 65°/sec, respectively) (Table 1). There was no significant correlation (r_right = 0.29, r_left = 0.22, *p* > 0.05) between stall speeds in BVD patients and their gains in lateral vHIT.

**Figure 3:**
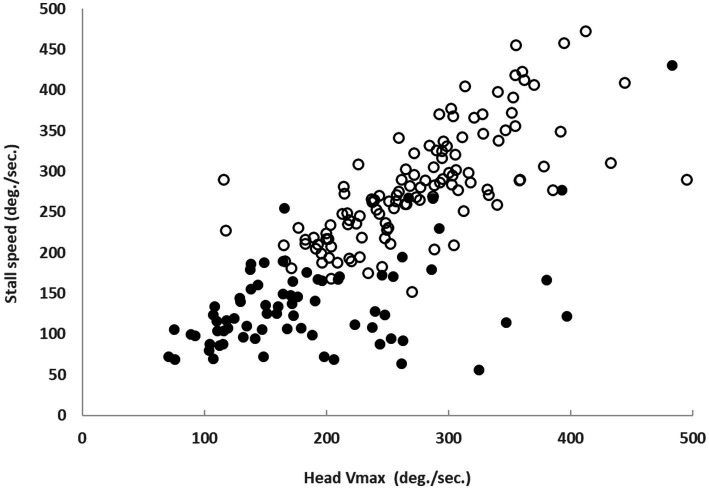
Correlation between spontaneous head velocity during Gaze-Shift and stall speed in 38 BVD patients (solid black dots, *r* = 0.51, *p* < 0.05) and 61 control subjects (white dots, *r* = 0.72, *p* < 0.05).

Stall speed was correlated with the head speed adopted spontaneously by control subjects (*r* = 0.72, *p* < 0.05) and most patients (*r* = 0.51, *p* < 0.05) (Figure 3). Maximum head speeds were significantly reduced (*p* < 0.01) in patients (mean ± SD: 186°/sec ± 79 to the right and 185°/sec ± 86 to the left) compared to control subjects (289°/sec ± 81 to the right and 268°/sec ± 64 to the left).

Gaze stabilization delays were influenced by head speed, saccade speed, and the need for corrective saccades during the counter-rotation phase in BVD patients. The right and left delays were 422 ± 109 ms and 404 ± 100 ms, respectively, significantly longer (*p*-right <0.01; *p*-left = 0.02) than in control subjects (349 ± 102 ms and 358 ± 63 ms, respectively).

BVD patients’ difficulties in stabilizing gaze during the counter-rotation phase led to a reduction in head movement amplitude during gaze shifts, resulting in a significant reduction (*p* < 0.01) in the head motion ratio (HMR) in patients (HMR right: 0.81 ± 0.25, HMR left: 0.77 ± 0.23) compared to control subjects (HMR right: 0.94 ± 0.22, HMR left: 1.03 ± 0.83). HMRs were significantly correlated with maximum head speeds (r_right = 0.62, r_left = 0.66) and stall speeds (r_right = 0.56, r_left = 0.45).

In contrast, the maximum speeds and amplitudes of eye saccades did not differ significantly between the two groups. None of the studied parameters showed a significant correlation with age.

**Figure 4:**
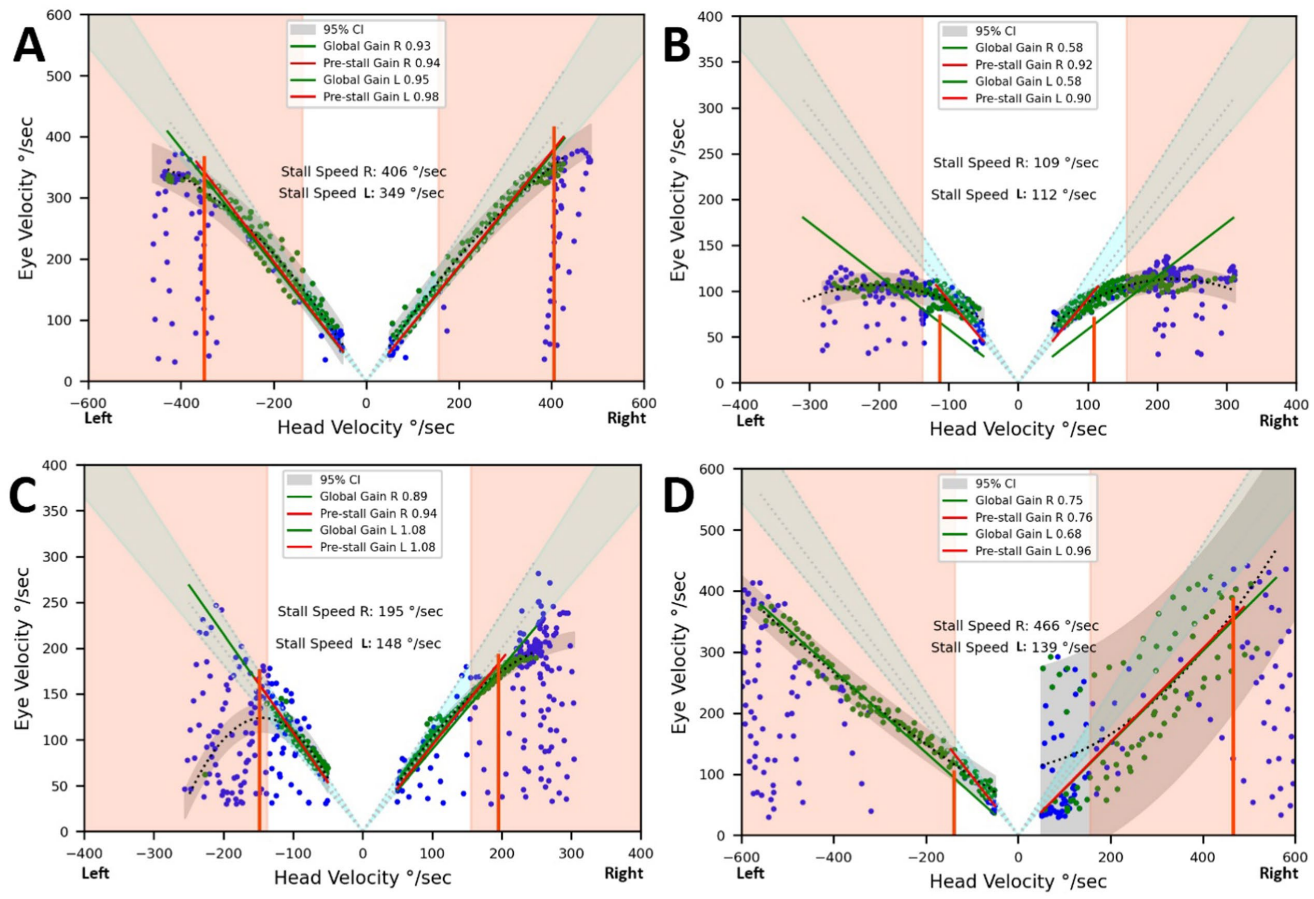
Eye and head velocities (degrees/second) during the counter-rotation phase. Vertical lines represent the stall speeds. The light grey areas indicate the normal range for overall gains and stall speeds. The dark grey area represents the prediction interval of the regression applied to the acquired data points. **(A)** Control subject. **(B)** Results from a patient diagnosed with CANVAS, showing head velocities limiting gaze stabilization to 109°/second on the right and 112°/second on the left. **(C)** Results from a patient with bilateral vestibular deficit (BVD) setting their own head rotation speed. Gaze stabilization was achieved up to 148°/second on the left (pre-stall gain and overall gain = 1.08) and 195°/second on the right (pre-stall gain = 0.94, overall gain = 0.89). **(D)** Same patient as in C, but instructed to turn their head as quickly as possible. This resulted in significantly degraded gaze stabilization, with an overall gain of 0.68 for leftward rotations and a wide variability in points on the right.

Figure 4 displays the relationships between eye and head velocities during the counter-rotation phase in a control subject (A), a BVD patient diagnosed with CANVAS (B), and a patient with idiopathic BVD when choosing their own head rotation speed (C) and when instructed to turn their head as quickly as possible (D). This resulted in significantly degraded gaze stabilization, with an overall gain of 0.68 for leftward rotations (for 1.08 with spontaneous head speed) and a wide variability in points on the right. When the head speed during gaze shifts exceeded the stall speed, gaze stabilization deteriorated, as reflected by the overall gain measurement. This impairment was also observed in four BVD patients who were asked to voluntarily increase their head speed beyond the spontaneously chosen speed (Figure 4D). The case illustrated shows a reduction in gain for leftward gaze rotation and greater variability for rightward movements. Figure 4B shows a situation where spontaneous head velocities exceed the stall speeds, which were limited to approximately 110°/sec. in a CANVAS patient.

## Discussion

Our results indicate that BVD patients spontaneously adopt a head speed during horizontal movements that significantly improves gaze stabilization during active movements compared to passive movements, as measured by vHIT. This improvement is evidenced by an average difference of 0.6 between the vHIT gain and the gaze-shift pre-stall gain for similar head speeds (Figure 2). Despite the enhancement in gaze stabilization, these mechanisms remain less effective in BVD patients compared to healthy subjects. The overall and pre-stall gains in BVD patients were significantly lower than those of control subjects and were associated with longer gaze stabilization times. Several mechanisms have been proposed to explain this improvement in performance during active head movements in BVD patients (8, 15–17).

**Figure 5:**
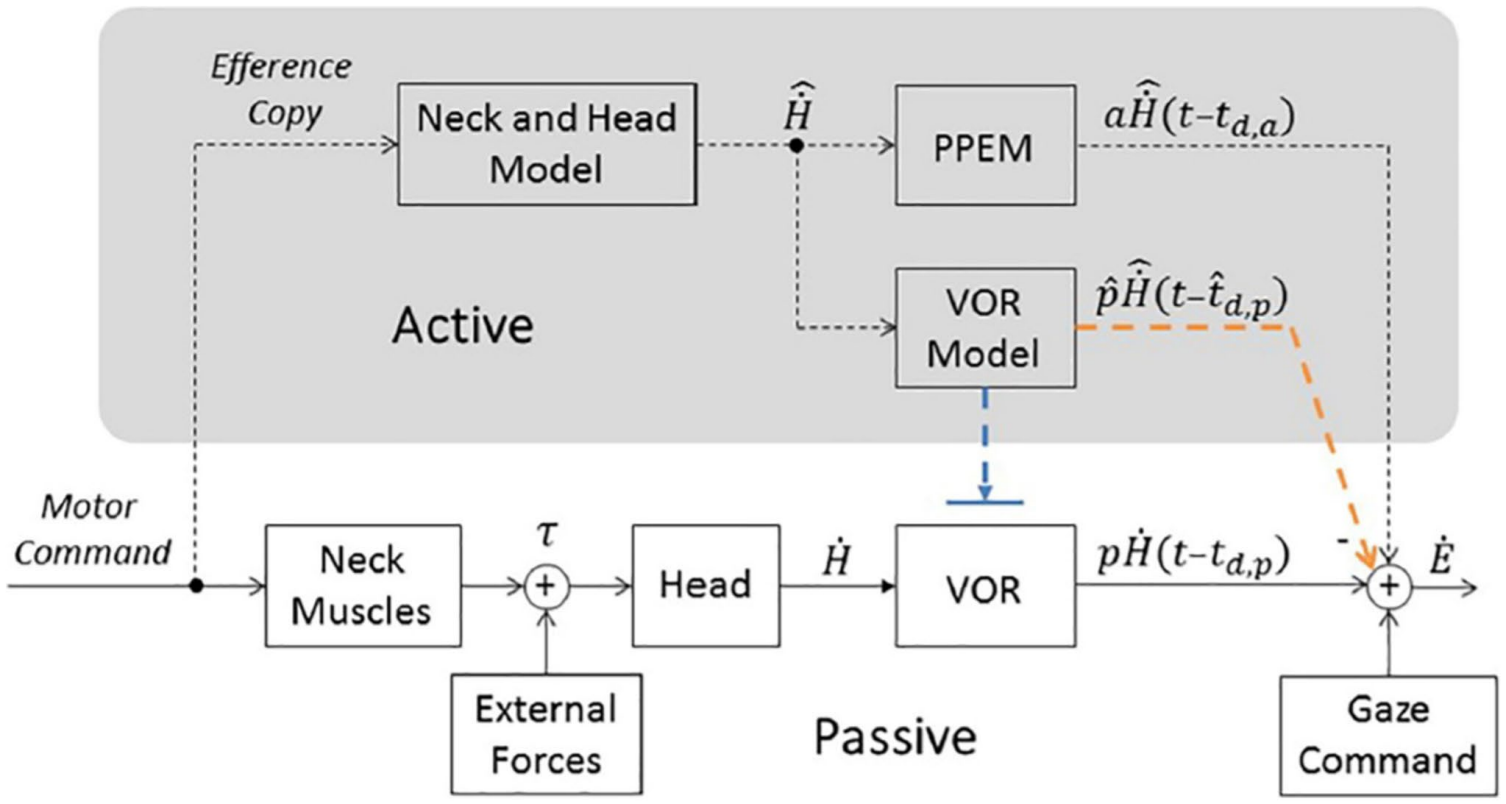
Model of gaze stabilization from Haggerty et al. (17). Bottom portion represents traditional pathways (i.e., the VOR and Gaze Command). Top portion (in gray, labeled “Active”) includes a pathway that estimates head velocity (“Neck and Head Model”) and necessary pre-programmed eye movements (“PPEM”) and two alternative pathways that interact with the VOR. The Suppression Model, in blue, that turns off the VOR and the Cooperative Model, in orange, that estimates the VOR’s response (“VOR Model”) and subtracts it from the total eye movement. H˙, Head velocity, p, passive gain, t_d,p_, passive time delay associated with the VOR pathway, Ė, Eye velocity, $\hat{\dot{H}}$ , prediction of the head velocity, a, gain of the PPEM, pˆ, estimation passive VOR gain, tˆ_d,p_, estimation of passive delay. Copyright © 2018 Haggerty and King. This is an open-access article distributed under the terms of the Creative Commons Attribution License (CC BY). The use, distribution or reproduction in other forums is permitted, provided the original author(s) and the copyright owner are credited and that the original publication in this journal is cited, in accordance with accepted academic practice.

In intact monkeys, position vestibular pause (PVP) neurons in the vestibular nuclei are the main interneurons for the VOR. Approximately 80% of these neurons receive strong monosynaptic input from the ipsilateral vestibular apparatus and project directly to contralateral extraocular motoneurons (22). They discharge in relation to eye and head velocity but cease firing during eye saccades. Figures 2B,C, 3 of reference (10) show that PVP neuron activity increases during the counter-rotation phase just after their pause during the initial eye saccade of gaze shifts in healthy monkeys. These authors demonstrated that the transmission of head velocity information from vestibular afferents to extraocular motoneurons by PVP neurons is reduced by approximately 60% during 60° active gaze shifts compared to the VOR during passive whole-body rotation. Nevertheless, in healthy humans and intact monkeys, gaze stabilization efficiency increases, with higher gains and shorter latencies during active head movements compared to passive ones (4, 23, 24). Unlike the gain reduction observed for the passive VOR from light to dark (23, 25), gaze stabilization performance remains equivalent under these two conditions for active gaze shifts (14). This has led to the proposal that VOR cooperates with feedback and feedforward mechanisms during the gaze stabilization phase of gaze shifts in intact subjects (8, 9, 14–17). Previous studies suggest that vestibular patients mainly rely on learned anticipatory mechanisms, rather than proprioception, to stabilize gaze during active head movements (8, 9, 15–17). Some observations appear contradictory: while Dichgans reported the absence of counter-rotation movement in intact monkeys during head blocking, suggesting an exclusive role for the VOR or a feedforward trigger, further increased gain after cervical deafferentation supports the hypothesis of pre-programmed eye movements (PPEM) (14). Thus, the exact contributions of VOR and PPEM to gaze stabilization during active head movements in intact beings remain to be clarified. A “cooperative model” (Figure 5) hypothesizes that the efference copy signal driving the PPEM is also used to predict and cancel eye movements that the VOR would produce in response to planned head movements (17). This would prevent the VOR from interfering with the PPEM while allowing it to compensate for unexpected head movements. This model seeks to reconcile previous experimental data by suggesting that PPEMs, in combination with VOR, are employed by both healthy and BVD subjects during active head movements.

However, the dynamics of this model must be adjusted according to the functional state of the vestibular system and head speed. If PPEM completely replaces the VOR for all speeds of active head movements in healthy subjects, there should be no difference between healthy and BVD patients when the VOR estimator has correctly integrated the chronic vestibular sensor deficit. Our results show, however, that BVD patients do stabilize their gaze but only at speeds lower than the stall speed. According to the “cooperative model, “insufficient eye speed beyond the stall speed could result either from difficulty in generating PPEMs at these speeds, or an erroneous prediction of the VOR deficit, or both. If the patient was capable of producing adequate eye movements during the counter-rotation phase before their vestibular deficit, and given that PPEM genesis is independent of vestibular sensors, there is no reason to hypothesize an inability to generate PPEMs for speeds above the stall speed. Instead, underestimation of the VOR deficit could lead to excessive inhibition of eye velocity during active movements. The differences observed in our BVD patients compared to control subjects might, therefore, result from an error in sensory prediction of the VOR deficit. This prediction error would gradually decrease after the onset of the deficit but would persist at speeds beyond the stall speed. A pre-stall gain close to 1 likely results from improved prediction of VOR function within the speed range below the stall speed. However, given the results of the caloric tests, it is unlikely that this improvement is due to enhanced VOR efficiency at lower speeds. The stall speed parameters and pre-stall gain would thus serve as indicators of the quality of sensory prediction of the vestibular deficit. The use of fixed visual targets could enhance prediction. The adaptation of the VOR prediction model to a VOR deficit occurs progressively. While it takes several months in monkeys, our unpublished recordings from subjects with acute unilateral vestibular deficits show that a pre-stall speed exceeding 100°/sec is achieved within a few weeks. Future studies should evaluate the effect of rehabilitation on the quality of this prediction. Vestibular rehabilitation could, by accelerating the head, improve sensory prediction for speeds above stall speed.

A repeated mismatch between sensory predictions and current sensory information, along with the symptoms it induces, might prompt the patient to reduce head speed to achieve greater accuracy in estimating the VOR deficit. The pre-stall speed could represent this optimal speed. Similarly, head movements in BVD patients may contribute to both targeting inaccuracy and discomfort, leading them to reduce the amplitude of head movement and increase ocular eccentricity during gaze shifts (8), as evidenced by the reduction in the head motion ratio observed in our study.

Figure 4B shows spontaneous head speeds (green dots reaching up to 300°/sec) exceeding stall speeds, around 110°/sec, in a CANVAS patient. This phenomenon is not observed in patients with isolated bilateral labyrinthine deficits and may reflect an inadequate internal model resulting from a motor learning deficit. Retinal slip at head velocities exceeding the stall speed does not appear to limit spontaneous head movement in this case. Isolated cerebellopathy does not impair gaze stabilization during gaze shifts (16), but combined with BVD could compromise the ability to predict VOR deficits and thus restrict his head velocity to stall speed. The integration of vestibular inputs with extravestibular self-motion signals in the vestibular cerebellum facilitates the computation of internal self-motion models, enabling coordinated eye, head, and body movements in response to expected and unexpected head motion changes (26).

In the patient illustrated (Figure 4D compared to Figure 4C), the requirement to turn the head as quickly as possible degrades performance on both sides. On the left side, gains decrease but remain consistent across recordings, while on the right, gains are more variable, showing significant dispersion. The horizontal VOR gains during vHIT for this patient are 0.64 on the right and 0.65 on the left for symmetrical head speeds between 200 and 300°/s. The observed asymmetry arises from differences in predictive capacities, which are mediated by distinct neural circuits for each side. Vestibular information is integrated with proprioceptive signals in the anterior vermis and with visual signals to generate internal models of head and eye movements, ensuring stable gaze (26). In the absence of asymmetric vestibular input, we hypothesize that asymmetry in predictive capacities may stem from differences in proprioceptive input, retinal split detection, or internal model implementation circuits.

Predictive coding is currently a leading theory of sensory information processing. Wade and Von Helmholtz (27) first argued that sensory inputs combine with our expectations to form the content of conscious experience (27). Current models propose that sensory perception relies on predictions rather than detailed information, and that our sensory experience constructs an explanation of the world (28). The brain’s task is to organize inputs into the most useful patterns, thereby reducing computational load by ignoring inputs that are unlikely to have adaptive value. This involves creating probabilistic models of the causes of current sensory inputs based on prior knowledge. These new models suggest that physical symptoms, as experienced and expressed by patients, do not directly result from sensor-derived information but from a mismatch between this and an inference based on implicit predictions derived from prior knowledge. This allows for a more nuanced understanding of the relationship between symptoms and peripheral sensor information, overcoming the artificial distinction between “explained” and “unexplained” physical symptoms (29). Symptoms may not originate from sensor dysfunction but from prediction error. Evaluating a subject’s sensory prediction performance in the context of vestibular symptoms is thus crucial not only for assessing compensation for a vestibular sensor deficit but also for understanding the emergence of vestibular symptoms in the absence of sensor dysfunction (19), such as in cases of vestibular migraines, PPPD, and anxiety-induced vertigo complaints (unpublished preliminary results).

In daily practice, gaze stabilization delay is a parameter rarely considered in the assessment of patients with vestibular deficits. In Maurer’s study of six patients, the total time to acquire a target at 60° was, on average, 780 ± 60 ms, compared to 467 ± 20 ms in healthy controls (8). The mean time to visually acquire targets in 31 space shuttle pilots immediately after landing was 7–10% greater than preflight values (30). Visual acuity and gaze stabilization efficiency have varying requirements depending on the task (31). For instance, the visual acuity needed when rotating the gaze to check for an oncoming vehicle before crossing a street is significantly less demanding than that required to read a bus number with the same head movement. Maximum visual acuity not only requires a stable image [retinal slip <4°/sec (32)] but also demands that the image is projected onto the most sensitive part of the retina for a sufficient duration. Loss of VOR efficiency or excessive phase shift moves the image away from the fovea. Each corrective saccade brings the image closer to the fovea, gradually increasing visual acuity (33). Patients with bilateral vestibular deficits may need up to four corrective saccades to focus on a visual target [Figure 1 from (25)]. As a result, full visual acuity is only restored after a delay, which can last several hundred milliseconds. This delay can be more disabling than image slippage on the retina during the 100 ms of head rotation. During the saccade phase of active movements, visual perception is suppressed and restored during the counter-rotation phase. The maximum allowable time for gaze stabilization in daily activities varies widely and can be demanding, such as when working with dual screens, driving, or engaging in sports (31, 34). Our BVD patients had significantly longer gaze stabilization times than control subjects. This delay resulted from slower head speeds and the presence of catch-up saccades (Figure 1B). This parameter appears useful for the functional assessment of patients with vestibular deficits. Preliminary results (not shown here) indicate that all these parameters can be used to monitor progress following acute unilateral vestibular deficit.

Previous studies and our results suggest that quality of life and oscillopsia questionnaires should distinguish between active predictable, passive predictable and passive unpredictable movements. Indeed, except in vehicles, many passive but repetitive movements can be anticipated (e.g., eye movements that anticipate head movements during locomotion) (35).

In summary, while measuring VOR in passive movement with vHIT is essential in the etiological diagnostic process, it seems insufficient for a comprehensive functional assessment of patients with vestibular deficits. Gaze stabilization performance is improved during active head movements, which are more common in daily life. We propose a simple vAGST to assess gaze stabilization performance during active spontaneous head movements. Our vAGST requires active and repetitive head-eye coordination, allowing us to investigate the capacity to develop predictive compensatory eye movements. Our results show that this efficiency varies depending on head speed in BVD patients. Despite a drastically reduced gain in the vHIT, our BVD patients demonstrated the ability to stabilize their gaze during active head movements at speeds averaging approximately 130°/sec. A single gain value is not sufficient to quantify gaze stabilization efficiency during active movements in vestibular deficit patients. We therefore propose to quantify gaze stabilization performance using stall speed, two gain values (pre-stall gain and overall gain), and gaze stabilization delay. The stall speed and pre-stall gain are not correlated with the gains measured by vHIT, and thus do not constitute redundant but complementary measurements reflecting different physiological mechanisms. This vAGST is not intended to provide an etiological diagnosis but rather a functional diagnosis based on the ability to develop predictive eye movements that compensate for vestibular sensor deficits. Access to information regarding the quality of the patient’s sensory predictions also provides a better understanding of symptoms such as dizziness and instability, even in the absence of a vestibular sensor deficit.

## Data Availability

The raw data supporting the conclusions of this article will be made available by the authors, without undue reservation.
